# *World Journal of Surgical Oncology* Reviewer Acknowledgement 2013

**DOI:** 10.1186/1477-7819-12-22

**Published:** 2014-01-31

**Authors:** Manoj Pandey

**Affiliations:** 1 Institute of Medical Sciences, BHU, Varanasi, India

## Abstract

**Contributing reviewers:**

The editor of *World Journal of Surgical Oncology* would like to thank all our reviewers who have contributed to the journal in Volume 11 (2013).

## 

Daniel Abbott

United States of America

Mohamed Abdel Wahab

Egypt

Mona Abdel-Hadi

Egypt

Taylor Abel

United States of America

Ghassan Abou-Alfa

United States of America

Miguel Abreu

Portugal

Mustapha Adham

France

Abbas Agaimy

Germany

Gaurav Agarwal

India

Amit Agrawal

Nepal

Archi Agrawal

India

Ritesh Agrawal

India

Youness Ahallal

Morocco

Wu Ai-Wen

China

Ichiro Akagi

Japan

Tomohiko Akahoshi

Japan

Bulent Akdogan

Turkey

Adenike Akhigbe

Nigeria

Tadashi Akiba

Japan

Shinsuke Akita

Japan

Luca Antonio Aldrighetti

Italy

Borislav Alexiev

United States of America

George Alexiou

Greece

Bilal Al-Jiffry

Saudi Arabia

Mohamed Farouk Allam

Spain

Majed Alokail

Saudi Arabia

Andrea Ambrosini-Spaltro

Italy

Maurizio Amichetti

Italy

Ali Aminian

Iran

Yasser Amr

Egypt

Nilakantan Ananthakrishnan

India

Benjamin Anderson

United States of America

John Anderson

United Kingdom

Koji Ando

Japan

Go Anegawa

Japan

Alessandro Antonelli

Italy

Melda Apaydin

Turkey

Giuseppe Aprile

Italy

Minoti V. Apte

Belize

Hossein Ali Arab

Iran

Vergilius Araujo-Filho

Brazil

Natarajan Aravindan

United States of America

Paul Arnold

United States of America

Vinod Arora

India

Jorge Arredondo

Spain

Nebojsa Arsenovic

United Kingdom

Christos Asteriou

Greece

Maurice Efana Asuquo

Nigeria

Evangelos Athanassiou

Greece

Mohammad Atif

Saudi Arabia

Rakototiana Auberlin

Madagascar

David August

United States of America

Paolo Aurello

Italy

Serhat Avcu

Turkey

Aïda Ayadi-Kaddour

Tunisia

Unal Aydin

Turkey

Miguel A. Ayerza

Argentina

Faisal Azam

United Kingdom

Negar Azarpira

Iran

Peter Baade

Australia

Flavia Baderca

Romania

Seong Kyu Baek

Korea South

Won-Jong Bahk

Korea South

Bilal Baker

Jordan

Marshall Baker

United States of America

Amanjit Bal

India

Saduman Balaban Adim

Turkey

Violetta Barbashina

United States of America

Nikolaos Barbetakis

Greece

Valeria Barresi

Italy

Richard Barth

United States of America

Erik Bärthel

Germany

David Bartlett

United States of America

Giancarlo Basili

Italy

Sandip Basu

India

Bahadir Batar

Turkey

Bilal Battal

Turkey

Shrujal Baxi

United States of America

Marek Bebenek

Poland

Kemal Behzatoglu

Turkey

Svein Ivar Bekkelund

Norway

Marcelo Beltran

Chile

Ehsen Ben Brahim

Tunisia

Idriss Benaiya Andaloussi

Morocco

Luigi Benecchi

Italy

Massimo Berger

Italy

Walter Berger

Austria

Martine Berliere

Belgium

Emilio Bertani

Italy

Adrian Billeter

Germany

Helgi Birgisson

Sweden

Debabrata Biswas

United Kingdom

Moises Blanco

Spain

Boris Blechacz

United States of America

Francesco Boccardo

Italy

Sylvie Bonvalot

France

Mahdi Bouassida

Tunisia

Souheil Boubia

Morocco

Jed Bouguila

Tunisia

Aldo Bove

Italy

Alexandre Bozec

France

Patrick Bradley

United Kingdom

Luka Brcic

Croatia

Todd Brickman

United States of America

Giuseppe Brisinda

Italy

Michael Bruneau

Belgium

Ansgar Brüning

Germany

Nicolas Buchs

Switzerland

Abdelbaset Buhmeida

Saudi Arabia

Jean Butte

Chile

Selim Buyukkurt

Turkey

Aman Buzdar

United States of America

Lu Cai

United States of America

José Cameselle-Teijeiro

Spain

Domenico Campanacci

Italy

Aras Emre Canda

Turkey

Liping Cao

China

Carlo Cappelli

Italy

Fabio Carboni

Italy

Sergio Cardoso

Brazil

Angelo Carretta

Italy

Andre Carvalho

Brazil

Riccardo Casadei

Italy

Vincenzo Catalano

Italy

Justin Cates

United States of America

Abigail Caudle

United States of America

Tom Cawood

New Zealand

Matteo Cescon

Italy

Karim Chahed

Tunisia

Chee-Yin Chai

Taiwan

Marc Chamberlain

United States of America

Kin Wai Edwin Chan

Hong Kong

Ruoh Shyuan Chan

Malaysia

Shirley Chan

United Kingdom

Coonoor Chandrasekar

United Kingdom

Hong Chang

China

Tsai-Wang Chang

Taiwan

Samuel Chao

United States of America

Grigoris Chatzimavroudis

Greece

Chih-Hao Chen

Taiwan

Chun-Hao Chen

United States of America

Sung-Lang Chen

Taiwan

Xiaoping Chen

China

Xiaowei Chen

United States of America

Zhouxun Chen

China

Wah Cheuk

Hong Kong

Nicolas Cheynel

France

Massimo Chiarugi

Italy

Akihiro Cho

Japan

Yun Ku Cho

Korea South

Joong Sub Choi

Korea South

Siu Ho Kenneth Chok

China

Danai Chourmouzi

Greece

Yong-Gu Chung

Korea South

James Church

United States of America

Fabio Cianchi

Italy

Saulius Cicenas

Lithuania

Halina Cichoz-Lach

Poland

Tommaso Cioppa

Italy

Gokhan Cipe

Turkey

Antonio Ciulla

Italy

Raphael Ciuman

Germany

Avram Cooperman

United States of America

Chiara Copelli

Italy

Fabio Coppedè

Italy

Fabio Corsi

Italy

Susan Costantini

Italy

Oana Craciunescu

United States of America

Marika Crohns

Finland

Linda Cummings

United States of America

Arnaud Dagain

France

Liping Dai

China

Zhijun Dai

China

Karina Danilowicz

Argentina

Dario D'Antonio

Italy

Giovanni Dapri

Belgium

Luigi De Cicco

Italy

Germano De Cosmo

Italy

Tommaso De Pas

Italy

Michele De Simone

Italy

Georgia Dedemadi

Greece

Marcos Dellaretti

Brazil

Elif Demirci

Turkey

Jing-Yu Deng

China

Kristoffer Derwinger

Sweden

Jean D'Haens

Belgium

Dipok Dhar

United Kingdom

Angelo Di Giorgio

Italy

Salomone Di Saverio

Italy

Rafael Díaz-Nieto

Spain

Song-Ze Ding

China

Xiangwu Ding

China

Joseph Disa

United States of America

Sanjay Dixit

United Kingdom

Mutlu Dogan

Turkey

Francesco Doglietto

Italy

Ahmet Bulent Dogrul

Turkey

Anna Domenech

Spain

Davide Maria Donati

Italy

Dimitrios Dragoumis

Greece

Jianjun Du

China

Peng Du

New Zealand

Guillaume Ducarme

France

Cecilia Duraes

Portugal

Polat Dursun

Turkey

L Dusek

Czech Republic

Janice Dutcher

United States of America

Konstantinos Economopoulos

United States of America

Ioannis Efthimiou

Greece

Susumu Eguchi

Japan

Behzad Einollahi

Iran

Konstantinos Ekmektzoglou

Greece

Magdy El Sherbiny

Egypt

Fatima Zahra Elfatoiki

Morocco

Ibrahim Elghissassi

Morocco

Mostafa Elhaddad

Egypt

Anthony Elias

United States of America

Luciana El-Kadre

Brazil

Prasad Ellanti

Ireland

Adam Elnaggar

United States of America

Ahmed Elzawawy

Egypt

Jean-Francois Emile

France

Fulop Emoke

Romania

Itaru Endo

Japan

Giorgio Ercolani

Italy

Costantino Errani

Italy

Andrew Fabiano

United States of America

Hong Fan

China

Hongbin Fan

China

Huizhi Fan

China

Yihui Fan

United States of America

Yujiang Fang

United States of America

Walid Faraj

Lebanon

Ahmad Faried

Indonesia

Luis Fariña

Spain

Mohammad Sadegh Fazeli

Iran

Luigi Fenoglio

Italy

Angel Fernandez-Flores

Spain

Daris Ferrari

Italy

Alessandro Ferrero

Italy

Kathryn Field

Australia

Joan Figueras

Spain

Ali Filiz

Turkey

Barbara Fischer

Denmark

Dominic Fong

Italy

Daniel Förnvik

Sweden

Christina Fotopoulou

United Kingdom

Joel Friedman

United States of America

Jianfei Fu

China

Bruno Fuchs

Switzerland

Hidenori Fujii

Japan

Shoichi Fujii

Japan

Takaaki Fujii

Japan

Takeo Fukagawa

Japan

Yosuke Fukunaga

Japan

Yasuhito Funahashi

Japan

Naotake Funamizu

Japan

Dominic Furniss

United Kingdom

Sunil V Furtado

India

Nazim Gümüş

Turkey

Wolfgang Gaertner

Mexico

Bertino Gaetano

Italy

Nicoletta Gagliano

Italy

Sun Gang

China

Jie Gao

China

Yang Gao

China

Joan Manel Gasent Blesa

Spain

Lukasz Gasiorowski

Poland

Tristan Gauthier

France

Bernhard Gebauer

Germany

Ralf Gertler

Germany

Sarbani Ghosh Laskar

India

Constantinos Giaginis

Greece

Zannoni Gian Franco

Italy

Maciej Giefing

Poland

Ziv Gil

Israel

Oliver Gimm

Sweden

Antonio Giorgio

Italy

Jacopo Giuliani

Italy

Paolo Giunta

Italy

Gabriel Glockzin

Germany

Ofer Gofrit

Israel

Murat Gokden

United States of America

Fernando Gómez

Spain

Adam Gondos

Germany

Javier Gonzalez

Spain

Lorena Gonzalez

Argentina

Augusto Gonzalvo

Australia

John Gore

United States of America

Gordan Grahovac

Croatia

Dan Granberg

Sweden

Gian Luca Grazi

Italy

John Griniatsos

Greece

Christina Gruber

Austria

Thomas Grunewald

France

Yan Gu

China

Tomas Gudbjartsson

Iceland

Felix Guerra-Gutierrez

Spain

Dorothy Gujral

United Kingdom

Ashish Gulia

India

Andrew Gumbs

United States of America

Guvem Gumus Akay

Turkey

Omer Gunal

Turkey

Orlando Guntinas-Lichius

Germany

Junming Guo

China

Juntang Guo

China

Wei Guo

China

Zhang Guo-Qing

China

Vikas Gupta

India

Richard Gurski

Brazil

Niraj Gusani

United States of America

Michael Haas

Germany

Oliver Haase

Germany

Zakaria Habib

Saudi Arabia

Vijay Hadda

India

Houssam Haddad

Morocco

Kenichi Hakamada

Japan

Samer Hakim

Germany

Michiyuki Hakozaki

Japan

Madoka Hamada

Japan

Kiyohiro Hamatani

Japan

Takeshi Hanagiri

Japan

Daniel Hänggi

Germany

Scott Hansen

United States of America

Torben B Hansen

Denmark

Noboru Hara

Japan

Georgia Hardavella

United Kingdom

Takahiro Hasebe

Japan

Kiyoshi Hasegawa

Japan

Suguru Hasegawa

Japan

Bassam Hassan

Malaysia

John Hayes

United Kingdom

Megan Haymart

United States of America

Jonathan Head

United States of America

Jose Ignacio Herrero

Spain

Tobias Hirsch

Germany

Vera Hirsh

Canada

Takeshi Hisa

Japan

Takeshi Hisa

Japan

Kwok-Ming Ho

Australia

Alan Hofmann

United States of America

Alan Hollingsworth

United States of America

Fy Hondo

United Kingdom

Gabriella Honeth

United Kingdom

Seok Jong Hong

United States of America

Naoko Honma

Japan

Tomohide Hori

United States of America

Marcus Horstmann

Germany

Terzah Horton

United States of America

Akihiro Hosaka

Japan

Chi-Hsun Hsieh

Taiwan

Miaofen Hu

United States of America

Xiang Hua

China

Chang-Ming Huang

China

Du Ping Huang

China

Gaosheng Huang

China

Martin Hübner

Switzerland

Philip Hylemon

United States of America

Cem Ibis

Turkey

Hisamitsu Ide

Japan

Hitoshi Ikeda

Japan

Osamu Ikeda

Japan

Shinichi Ikuta

Japan

Andrea Imperatori

Italy

Ryo Inada

Japan

Ugo Indraccolo

Italy

Pilar Iniesta

Spain

Abid Irshad

United States of America

Mitsuaki Ishida

Japan

Toru Ishikawa

Japan

Nabil Ismaili

Morocco

Yukihiro Iso

Japan

Osamu Itano

Japan

Hideaki Ito

Japan

Yasuhiro Ito

Japan

Yasuo Iwadate

Japan

Hirotaka Iwase

Japan

Hasnan Jaafar

Malaysia

Mehdi Jaidane

United Kingdom

Faek Jamali

Lebanon

Tae Jung Jang

Korea South

Omer Janjua

Pakistan

Julien Jarry

France

Daiva Jasaitiene

Lithuania

Jiafu Ji

China

Hao Jiang

China

Shujun Jiang

China

Wenjie Jiao

China

Montiel Jimenez Fuertes

Spain

Heikki Joensuu

Finland

Elio Jovine

Italy

Passas Juan

Spain

Fevziye Kabukcuoglu

Turkey

Dariusz Kaczmarczyk

Poland

Erdinc Kamer

Turkey

Estela Kaminagakura

Brazil

Terumi Kamisawa

Japan

Makoto Kammori

Japan

Hironori Kaneko

Japan

Chang Hyun Kang

Korea South

Murat Kapan

Turkey

Edna Kapenhas-Valdes

United States of America

Riken Karachi

Japan

Guldeniz Karadeniz

Turkey

Elias Karakas

Germany

Omur Karakoyun Celik

Turkey

Jose Karam

United States of America

Ayse Karaman

Turkey

Steven Katz

United States of America

Dara Kavanagh

Ireland

Junichiro Kawamura

Japan

Mahmood Kayani

Pakistan

Airazat Kazaryan

Norway

Mehmet Kefeli

Turkey

Ilknur Kepenekci

Turkey

Avi Khafif

Israel

Jim Khan

United Kingdom

Rahul Khanna

India

Joon Joon Khoo

Malaysia

Piya Kiatisevi

Thailand

Yuko Kijima

Japan

Eiji Kikuchi

Japan

Luciana Kikuchi

Brazil

Jonghan Kim

Korea South

Sunghoon Kim

Korea South

Young Tae Kim

Korea South

Gretchen Kimmick

United States of America

Takuya Kimura

Japan

Akiyoshi Kinoshita

Japan

Hiroyuki Kirikoshi

Japan

Boris Kirshtein

Israel

Sohei Kitazawa

Japan

Aarne Kivioja

Finland

Tobias Klatte

Austria

Shigeru Kobayashi

Japan

Oguz Koc

Turkey

Claus Koelblinger

Austria

Fumitaka Koga

Japan

Kamal Koirala

Nepal

Masaru Koizumi

Japan

Triantafyllia Koletsa

Greece

Virve Koljonen

Finland

Shuhei Komatsu

Japan

Eiichi Konishi

Japan

Vasileios Kontogeorgakos

Greece

Katerina Kontogianni

Greece

Dimitris Korkolis

Greece

Gabriela Kornek

Austria

Christoforos Kosmidis

Greece

Georgios Koukourakis

Greece

Chryssanthos Kouriefs

Cyprus

Georgios Koutalellis

Greece

Avdyl Krasniqi

Serbia

Yujin Kudo

Japan

Julita Kulbacka

Poland

Koji Kumagai

Japan

Neeta Kumar

India

Vinita Kumar

India

Liu Kun

China

Chikara Kunisaki

Japan

Yur-Ren Kuo

Taiwan

Serhan Küpeli

Turkey

Markus Küper

Germany

Toru Kusano

Japan

Toshihiro Kushibiki

Japan

Tae-Won Kwon

Korea South

Eric C.H. Lai

Hong Kong

Nirmal Lamichhane

Nepal

Alessandro Landi

Italy

Hauke Lang

Germany

Andreas Larentzakis

Greece

Francesco Latrofa

Italy

Joseph W.Y. Lau

Hong Kong

Patrick Lau

Hong Kong

Seung-Hyun Lee

Korea

Yoon-Suk Lee

Korea

Katia Leite

Brazil

Frank Lenze

Germany

Benjamin Leong

Malaysia

Edmund Leung

United Kingdom

Chung-Pin Li

Taiwan

Hui Li

China

Jianmin Li

China

Rachel Li

Australia

Songtao Li

China

Zhi Li

China

Zhenyu Liang

China

Justus Lieber

Germany

David Lieu

United States of America

Che Lin

Taiwan

Jen-Der Lin

Taiwan

Zhenhua Lin

China

Dimitrios Linos

Greece

Julius Liptak

Canada

Caigang Liu

China

Jiangang Liu

United States of America

Jie Liu

United States of America

Ning Liu

China

Tian-Run Liu

China

Varut Lohsiriwat

Thailand

Ambrogio Pietro Londero

Italy

Rafael Lopez- Andujar

Spain

Pia Lopez Jornet

Spain

Antonio Lopez-Beltran

Spain

Julio Lopez-Monclova

Mexico

Jan-Christoffer Luers

Germany

Carmelo Luigiano

Italy

Alberto Luini

Italy

Yongchao Ma

China

Marcel Autran Cesar Machado

Brazil

Norman Machado

Oman

Laszlo Madacsy

Hungary

Daichi Maeda

Japan

Yoshihiko Maehara

Japan

Hiroshi Maekawa

Japan

Stefano Magno

Italy

Magdalena Magnowska

Poland

Bilal Majed

France

Geert Maleux

Belgium

Aljosa Mandic

Serbia

Spilios Manolakopoulos

Greece

Varsha Manucha

United States of America

Nicoletta Maounis

Greece

Roberto Marcen-Letosa

Spain

Jacques Marescaux

France

Fernanda Mariano

Brazil

Athanasios Marinis

Greece

Ugo Marone

Italy

Christine Marosi

Austria

Daniele Marrelli

Italy

Jacopo Martellucci

Italy

Jeremiah Martin

United States of America

Jeremiah Martin

United States of America

Satoshi Maruyama

Japan

Michele Masetti

Italy

Michele Masetti

Italy

Suresh Masilamani

India

Ashiq Masood

United States of America

Ashiq Masood

United States of America

Rohit Mathur

India

Masanori Matsuda

Japan

Takeshi Matsutani

Japan

Majid Maybody

United States of America

Lucas Mccormack

Argentina

Michael Mcfarlane

Jamaica

Joshua Meeks

United States of America

Asadi Mehrnaz

Iran

Eiji Mekata

Japan

Ehud Mendel

United States of America

Rudolf Mennigen

Germany

Rudolf Mennigen

Germany

Francesco Meriggi

Italy

George Metaxas

United Kingdom

Philippe Metellus

France

Caterina Mian

Italy

Michael Michael

Australia

Shuji Mikami

Japan

Zsofia Miltenyi

Hungary

Thomas Miner

United States of America

Jan Mintorovitch

United States of America

Massimiliano Mistrangelo

Italy

Yasuhiro Miyake

Japan

Masaru Miyazaki

Japan

Shinjiro Mizuguchi

Japan

Toru Mizuguchi

Japan

Simone Mocellin

Italy

Hitesh Modi

India

Helmout Modjtahedi

United Kingdom

Yasuhiko Mohri

Japan

Ghadeer Mokhtar

Saudi Arabia

Michele Molinari

Canada

Ali Montazeri

Iran

Rodolfo Montironi

Italy

Katherine Moore

Canada

Umberto Morelli

France

Paolo Morgagni

Italy

Takeshi Morii

Japan

Ken Morita

Japan

Gareth Morris-Stiff

United Kingdom

Hisham Mosli

Saudi Arabia

Khouchani Mouna

Morocco

Giannis Mountzios

France

Guillaume Mousseau

United States of America

Andre Mueller

United States of America

Oliver Mueller

Germany

Sven Mueller

Germany

Anja Mühlfeld

Germany

Francisco Cristóbal Muñoz-Casares

Spain

Andrea Muratore

Italy

Jane Muret

France

Claudio Murta

Brazil

Giuseppe Musumeci

Italy

Rakesh Naidu

Malaysia

Tohru Nakagawa

Japan

Ryoichi Nakanishi

Japan

Yoshifumi Nakao

Japan

Satoshi Nakasu

Japan

Atsushi Nambu

Japan

Anil Nanda

United States of America

Kannan Nar

India

Angelo Naselli

Italy

Rachael Natrajan

United Kingdom

Christopher Neal

United Kingdom

Sonia Nemolato

Italy

Alessandro Neri

Italy

Romana Netea-Maier

Netherlands

Simon Ng

Hong Kong

Phan Nguyen

Australia

Carsten Nieder

Norway

Giuseppe Nigri

Italy

Raje Nijhawan

India

Parvaneh Nikpour

Iran

Yuji Nimura

Japan

Reiki Nishimura

Japan

Jun Nishio

Japan

Satoshi Nishizuka

Japan

Hiroyuki Nitta

Japan

Emeka Nkenke

Germany

Mario Nosotti

Italy

Ewa Nowakowska-Zajdel

Poland

Peter Nthumba

Kenya

Evarist Tm Nyawawa

Tanzania

Gerardo Andres Obeso Carillo

Spain

Mark O'donnell

United States of America

Tadanori Ogata

Japan

Hiroshi Ogawa

Japan

Shuji Ogino

United States of America

Yoshio Ohno

Japan

Koichi Oishi

Japan

Hidenori Ojima

Japan

Jiro Okami

Japan

Tomohisa Okuma

Japan

Masaya Okura

Japan

Ramesh Omranipour

Iran

Nilufer Onak Kandemir

Turkey

Satoshi Ono

Japan

Nada Orsolic

Croatia

Clement Osime

Nigeria

Javier Osorio

Spain

Sharon O'toole

Ireland

Takaharu Oue

Japan

Bala Basak Oven Ustaalioglu

Turkey

Shinji Ozaki

Japan

Vahit Ozmen

Turkey

Fabio Pagni

Italy

Rama Pai

United States of America

Chin-Chen Pan

Taiwan

Durgatosh Pandey

India

Nikolaos Papadopulos

Germany

David pda

Venezuela

Dhavan Parikh

United States of America

Rojin Park

Korea South

Grace Parkins

Ghana

Tomas Paseka

Czech Republic

Subroto Paul

United States of America

Nicholas Pavlidis

Greece

Kalliopi Pazaitou-Panayiotou

Greece

Corrado Pedrazzani

Italy

Fei Pei

China

Luke Peppone

United States of America

Stephanos Pericleous

United Kingdom

Marcos Perini

Brazil

Jan Persson

Sweden

Christopher Pezzi

United States of America

Lynette Phillips

United States of America

Antonio Piñero

Spain

Patorn Piromchai

Thailand

Francesco Pisani

Italy

Chiara Adriana Pistolese

Italy

Poramate Pitak-Arnnop

Germany

Michael Pitiakoudis

Greece

Henry Pitt

United States of America

Benjamin Piura

Israel

Stefania Pizzimenti

Italy

Przemyslaw Plonka

Poland

Francesco Polistina

Italy

Marco A Ponce-Camacho

Mexico

Loukia Poulou

Greece

Stephen P. Povoski

United States of America

Chris Protzel

Germany

Kyriakos Psarras

Greece

Julian Puppe

Germany

Peng Qi

China

Felisbina Queiroga

Portugal

Roy Raad

United States of America

Marius Raica

Romania

Lois Ramondetta

United States of America

Roshni Rao

United States of America

Shahnawaz Rasheed

United Kingdom

Ib Rasmussen

Sweden

Maria Rosaria Raspollini

Italy

Ruchi Rastogi

India

Sadykov Rasul

Uzbekistan

Chandrajit Raut

United States of America

Federico Rea

Italy

Maximino Redondo

Spain

Mohamed Regal

Saudi Arabia

Leonardo Reis

Brazil

Bharat Rekhi

India

Zhenggang Ren

China

Carla Ribeiro

Portugal

Mohammed Ridai

Morocco

Marc Riquet

France

Jesús Rivera-Navarro

Spain

Fabio Roberti

United States of America

Stuart Michael Robinson

United Kingdom

Javier Rodriguez

Spain

Simon Rogers

United Kingdom

Moshe Rogosnitzky

Israel

Jong-Lyel Roh

Korea South

Daniel Rojas

Bolivia

Nana Rokutanda

Japan

Fabrizio Romano

Italy

Ferdinando Rombola

Italy

Ulrich Ronellenfitsch

Germany

Robert Rosenberg

Switzerland

Isabel Rubio

Spain

Natale Russo

Italy

Piotr Rutkowski

Poland

Andrea Ruzzenente

Italy

Min-Hee Ryu

Korea South

Primuharsa Putra S H A

Malaysia

Alan Saber

United States of America

Aly Saber

Egypt

Virgilio Sacchini

United States of America

Maha Safwat

Egypt

Birch Saheeb

Nigeria

Kabul Saikia

India

Hidetsugu Saito

Japan

Mitsue Saito

Japan

Yasushi Sakamaki

Japan

Akio Sakamoto

Japan

George Sakorafas

Greece

Nikolaos Salemis

Greece

Milan Samardjiski

Macedonia

Paolo Sammartino

Italy

Alvaro Sanabria

Colombia

Sakiko Sanada

Japan

Mario Sanna

Italy

Luigi Santambrogio

Italy

Mario Santinami

Italy

Daniele Santini

Italy

Mario Santini

Italy

Alain Saraux

France

Andrea Sardella

Italy

Gokhan Sargin

Turkey

Devanand Sarkar

United States of America

Inderpal Sarkaria

United States of America

Sergio Sartori

Italy

Akira Sasaki

Japan

Ken Sasaki

Japan

Ken Sasaki

Japan

Sith Sathornsumetee

Thailand

Hiroaki Satoh

Japan

Yukitoshi Satoh

Japan

Sohei Satoi

Japan

Alain Sauvanet

France

Niramol Savaraj

United States of America

Svetlana Savin

Serbia

Noriyoshi Sawabata

Japan

Shigeki Sawada

Japan

Stefano Scabini

Italy

Mario Scartozzi

Italy

Niklaus Schaefer

Switzerland

Katharina Schmid

Germany

Sven-Christian Schmidt

Germany

Joachim Schmutzhard

Austria

Berit Schneider-Stickler

Austria

Justine Schober

United States of America

Sebastian F. Schoppmann

Austria

Arjan Schouten Van Der Velden

Netherlands

David Schreiber

United States of America

Carsten Schroeder

United States of America

Halil Ibrahim Seçer

Turkey

Fernanda Seixas Travassos

Portugal

Lorenzo Sempere

United States of America

Alex Senchenkov

United States of America

Theodoros N. Sergentanis

Greece

José Mª Serra-Renom

Spain

Yasuyuki Seto

Japan

Wael Sewilam

Egypt

Abeer Shaaban

United Kingdom

Ali Shamaa

Egypt

Suresh Sharma

United States of America

Shyr-Ming Sheen-Chen

Taiwan

Lizong Shen

China

Ming Shi

China

Zhong-Song Shi

China

Hiroaki Shiba

Japan

Chikashi Shibata

Japan

Alexander Shifrin

United States of America

Daphne Shih Wen

Singapore

Yoshihisa Shimada

Japan

Toru Shimazui

Japan

Shoji Shimoyama

Japan

Takehiko Shimoyama

Japan

Takaya Shimura

Japan

Dong Hoon Shin

Korea South

Hisashi Shinohara

Japan

Toshihiko Shinohara

Japan

Masaru Shinozaki

Japan

Kuniaki Shirao

Japan

Shailesh Shrikhande

India

Vijay Shukla

India

Robert Siegel

Germany

Llewellyn Sim

Singapore

Nektaria Simiantonaki

Germany

Giuseppe Simone

Italy

Virendra Singh

India

Tristan Sissung

United States of America

Pavel Skok

Slovenia

Natalija Snovak

Croatia

Luciano Solaini

Italy

Gaztambide Sonia

Spain

Makoto Sonobe

Japan

Cristina Sottani

Italy

Mehdi Soufi

Morocco

Petros Sountoulides

Greece

Cosimo Sperti

Italy

Claudio Spinelli

Italy

Maria Silvia Spinelli

Italy

Salvatore Squillaci

Italy

Bart Staels

France

Konstantinos Stamatiou

Greece

Giorgio Stassi

Italy

Justin Stebbing

United Kingdom

Sonja E. Steigen

Norway

Eberhard Stoeckle

France

Sandro Stoeckli

Switzerland

Zorica Stojsic

Serbia

Christian Sturesson

Sweden

Rongjian Su

China

Xiaodong Su

China

Daryl Subramaniam

United Kingdom

Subramanian Subramanian

United States of America

Takeshi Sudo

Japan

Iwao Sugitani

Japan

Laurent Sulpice

France

Aziz Sumer

Turkey

Shibin Sun

China

Anil Suri

India

Tommaso Susini

Italy

Shuji Suzuki

Japan

Shuji Suzuki

Japan

Lars Bo Svendsen

Denmark

Jeff J Swensen

Australia

Vlasta Sykorova

Czech Republic

John Syrios

United Kingdom

Yuji Tachimori

Japan

Keiichiro Tada

Japan

Deepa Taggarshe

United Kingdom

Luca Tagliabue

Italy

Kazuaki Takabe

United States of America

Ryo Takagawa

Japan

Hiroki Takahashi

Japan

Mamoru Takahashi

Japan

Masashi Takano

Japan

Satoru Takayama

Japan

Tomoyoshi Takenaka

Japan

Hiroya Takeuchi

Japan

Nagio Takigawa

Japan

Shigeyuki Tamura

Japan

Katsuhiro Tanaka

Japan

Koji Tanaka

Japan

Kuniya Tanaka

Japan

Nobumichi Tanaka

Japan

Liming Tang

China

Siriwan Tangjitgamol

Thailand

Nobuhiko Tanigawa

Japan

Shinya Tanimura

Japan

Luciano Tarantino

Italy

Shoh Tatebe

Japan

Georgi Tchernev

Bulgaria

Julia Tchou

United States of America

Timo Ten Hagen

Netherlands

Melissa Teo

Singapore

Koji Teramoto

Japan

Satoshi Teramukai

Japan

Massimo Terzolo

Italy

Hardik Thakker

India

George Theodoropoulos

Greece

Khin Thway

United Kingdom

Xavier Tillou

France

Kazunori Tobino

Japan

Yasushi Toh

Japan

Moustapha Tohfe

Lebanon

Gokhan Toktas

Turkey

Dario Tomasini

Italy

Qiangsong Tong

China

Ursula Tonnhofer

Austria

Vladislav Treska

Czech Republic

Pierpaolo Trimboli

Italy

Pierpaolo Trimboli

Italy

Mounir Trimeche

Tunisia

Dragan Trivanovic

Croatia

Priti Trivedi

India

Mark Trombetta

United States of America

Gary Tse

Hong Kong

Hironori Tsujimoto

Japan

Kazunori Tsukuda

Japan

Kazunori Tsukuda

Japan

Toshio Tsuyuguchi

Japan

Shuiping Tu

China

Raymond Tubbs

United States of America

Mehmet Turgut

Turkey

Todd Tuttle

United States of America

James Tysome

United Kingdom

Ching Tzao

Taiwan

Junji Ueda

Japan

Takahiro Uenishi

Japan

Cigdem Ulukaya Durakbasa

Turkey

Diclehan Unsal

Turkey

Pradeep Vaideeswar

India

J. Fernando Val-Bernal

Spain

Sniya Valsa Sudhakar

India

Anton Van Lierop

South Africa

Manish Varshney

India

Yusuf Vayisoglu

Turkey

Gregory Velat

United States of America

Tomaz Velnar

Slovenia

Frederic Venail

France

Frederik Verburg

Germany

Giulia Veronesi

Italy

Gustavo Viani

Brazil

Ramon Vilallonga

Spain

B Viswanatha

India

Gonzalo Vitagliano

Argentina

Christian Von Falck

Germany

Margaret Von Mehren

United States of America

Camelia Doina Vrabie

Romania

Tze Wah

United Kingdom

Roger Wahba

Germany

Toshifumi Wakai

Japan

Jennifer Waljee

United States of America

Michael Wallace

United States of America

Shutao Wang

United States of America

Xishan Wang

China

Xishan Wang

China

Yuehua Wang

China

Imtiaz Wani

India

John Ward

United States of America

Atsushi Watanabe

Japan

Kazuhiro Watanabe

Japan

Christina Westhoff

Germany

Jaidev Wig

India

Philipp Wiggermann

Germany

Johan Wiig

Norway

Helmut Wilhelm

Germany

Dillwyn Williams

United Kingdom

Andrzej Wincewicz

Poland

Beate Winkler

Germany

James Stuart Wolf

United States of America

Thian-Sze Wong

Hong Kong

Jie Wu

China

June Wu

United States of America

Liang Wu

China

Yu-Chung Wu

Taiwan

Hongping Xia

China

Yanqun Xiang

China

Gui-Zhou Xiao

China

Mingzhao Xing

United States of America

Kazutaka Yamada

Japan

Yoshiaki Yamagata

Japan

Hidetaka Yamamoto

Japan

Masakazu Yamamoto

Japan

Norio Yamamoto

Japan

Takumi Yamamoto

Japan

Takahiro Yamasaki

Japan

Hiroko Yamashita

Japan

Yo-Ichi Yamashita

Japan

Makoto Yamauchi

Japan

Zhu Yang

China

Tomoyuki Yano

Japan

Takahiro Yasui

Japan

Bin Yi

China

Yusuf Yildirim

Turkey

Linwah Yip

United States of America

Kohei Yokoi

Japan

Takaki Yoshikawa

Japan

Seiichi Yoshimoto

Japan

Kunio Yoshizawa

Japan

Haney Youssef

United Kingdom

Guang-Yan Yu

China

Tinghe Yu

China

Zhong-Yu Yuan

China

Young Jin Yuh

Korea South

Ohtsuki Yuji

Japan

Hyun Don Yun

United States of America

Sohila Zadran

United States of America

Carles Zafon

Spain

Olaf Zagólski

Poland

Nicola Zampieri

Italy

Smart Zeidan

Lebanon

Bernhard Zelger

Austria

Zhao-Chong Zeng

China

Fukui Zhang

China

Honzi Zhang

China

Jin Zhang

China

Qixu Zhang

United States of America

Sen Zhang

China

Wei-Dong Zhang

China

Hua-Chuan Zheng

China

Jun-Hua Zheng

China

Lei Zheng

United States of America

Liduan Zheng

China

Shusen Zheng

China

Shusen Zheng

China

Chuan-Xiang Zhou

China

Li Zhou

China

Ping-Hong Zhou

China

Jin-Shui Zhu

China

Nina Zidar

Slovenia

Oded Zmora

Israel

Giovanni Zoccali

Italy

